# Venous thromboembolism and COVID-19: a case report and review of the literature

**DOI:** 10.1186/s13256-020-02516-4

**Published:** 2020-10-15

**Authors:** Harshil Bhatt, Sandeep Singh

**Affiliations:** 1 Goshen Health Hospital, 200 High Park Ave, Goshen, IN 46526 USA; 2 Indiana University School of Medicine, 1234 N Notre Dame Ave, South Bend, IN 46617 USA

**Keywords:** COVID-19, Venous thromboembolism, Pulmonary embolism, Sars-CoV-2

## Abstract

**Background:**

Currently, there is minimal data available highlighting the prevalence of venous thromboembolism in patients infected with coronavirus disease 2019 (COVID-19). This case report with a literature review emphasizes a unique presentation of COVID-19 that is highly important for health care providers to consider when treating their patients.

**Case report:**

A 65-year-old Caucasian male patient presented to the emergency department with a 2-day history of dyspnea on exertion after his wife’s recent diagnosis of COVID-19. He additionally had experienced a couple of episodes of self-resolving diarrhea a few days before presentation. Based on the patient’s clinical presentation and the laboratory workup identifying an elevated D-dimer, a computed tomography angiogram of the chest was obtained, which was significant for moderately large, bilateral pulmonary emboli with a saddle embolus, and an associated small, left lower lobe, pulmonary infarct. Ultrasound of the lower extremity showed non-occlusive deep vein thrombosis at the distal left femoral vein to the left popliteal vein. The patient was additionally diagnosed with COVID-19 when the results of the COVID-19 polymerase chain reaction test returned as positive. The patient was admitted to the COVID unit, and he was started on an intravenously administered, unfractionated heparin drip for management of his bilateral pulmonary emboli and deep vein thrombosis. The patient’s clinical condition improved significantly with anticoagulation, and he was observed in the hospital for 3 days, after which he was discharged home on the enoxaparin bridge with warfarin. Post-discharge telephone calls at day 10 and week 4 revealed that the patient was appropriately responding to anticoagulation treatment and had no recurrence of his symptoms related to venous thromboembolism and COVID-19.

**Conclusion:**

As COVID-19 continues to lead to significant mortality, more data is emerging that is exposing its perplexing pathogenicity. Meanwhile, the presentation of venous thromboembolism in patients with COVID-19 remains an unusual finding. It is imperative for health care providers to be mindful of this unique association to make necessary diagnostic evaluations and provide appropriate treatment for the patients.

## Introduction

The coronavirus disease 2019 (COVID-19) pandemic, caused by severe acute respiratory syndrome coronavirus-2 (SARS-CoV-2), continues to desolate a significant portion of the world’s population, and health care providers continue to see new and frightening displays of its pathogenicity. We report an atypical case involving a 65-year-old male patient with an acute saddle pulmonary embolism and a deep vein thrombosis (DVT) associated with COVID-19.

### Case report

A 65-year-old Caucasian male patient presented to the emergency room with a 2-day history of dyspnea on exertion. He also noted experiencing a couple of episodes of diarrhea a few days before his dyspnea started, which resolved on its own. The patient’s wife had recently been diagnosed with COVID-19. The patient denied any fever, cough, chest pain, or lower-extremity edema. His past medical history included type 2 diabetes, hypertension, and hyperlipidemia. His past surgical history included a remote history of arthroscopic knee surgery. No personal history of malignancy was noted. No family history of hypercoagulable disease or thromboembolism was present. The patient had never smoked, denied drinking alcohol, and had previously been taking metformin, amlodipine, and simvastatin when at home.

**Fig. 1:**
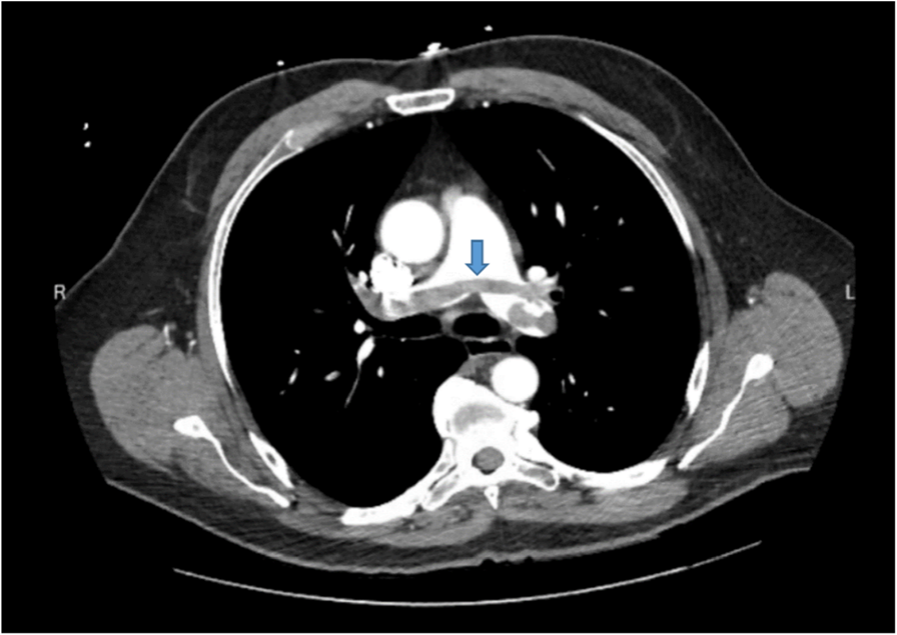
Computed tomography (CT) angiogram of the chest showing large, bilateral pulmonary emboli with a saddle embolus (blue arrow)

**Fig. 2:**
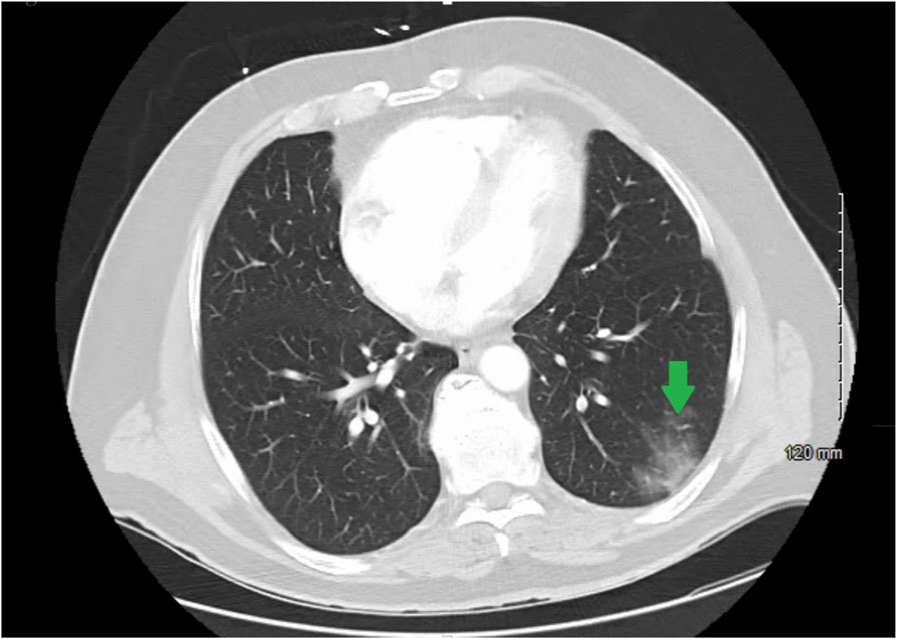
Computed tomography (CT) angiogram of the chest (lung window) showing an evolving small, left lower lobe, pulmonary infarct (green arrow)

His vital signs included blood pressure of 150/98 mmHg, pulse rate of 97 beats/min, respiratory rate of 18/min, oximetry 97% on room air, and a temperature of 36.5 °C (97.8 °F). Pertinent findings on physical examination included clear breath sounds and a regular, rapid heart rhythm on auscultation. There was no lower-extremity edema or calf tenderness. An electrocardiogram (EKG) completed in the emergency room showed sinus tachycardia at a rate of 113 bpm with no acute ST-T segment changes. A chest x-ray showed no acute cardiopulmonary findings. Laboratory data showed a white count of 6.9 × 10^9^/L, hemoglobin of 15.3 g/dL, hematocrit at 44.9%, and platelet count of 158 × 10^9^/L. The basic metabolic panel was within the normal range except for the blood sugar of 235 mg/dL. Troponin was 0.16 ng/mL, with normal total creatinine kinase. D-dimer was elevated at 2241 ng/mL (Table 1). The routine respiratory polymerase chain reaction (PCR) panel was negative. A SARS-CoV-2 PCR test was sent from the emergency room. A computed tomography (CT) angiogram of the chest showed moderately large, bilateral pulmonary emboli with a saddle embolus and evidence of slight right heart strain and pulmonary arterial hypertension with an associated small, left lower lobe, pulmonary infarct (Figs. 1 and 2).

**Table 1:** Laboratory data

Laboratory test	Results	Reference range
White blood cell count	6.9 × 10^9^/L	3.9–10.5 × 10^9^/L
Red blood cell count	4.9 × 10^12^/L	4.33–5.73 × 10^12^/L
Hemoglobin	15.3 g/dL	13.6–17.0 g/dL
Hematocrit	44.9%	40.0–54.0%
Platelet count	158 × 10^9^/L	150–450 × 10^9^/L
Prothrombin	13.1 s	10.68–13.72 s
INR	1.1	
D-dimer	**2241** ng/mL	0–253.5 ng/mL
Sodium	138 mmol/L	137–145 mmol/L
Potassium	4.5 mmol/L	3.4–5.1 mmol/L
Chloride	103 mmol/L	98–107 mmol/L
Carbon dioxide	23 mmol/L	22–30 mmol/L
Anion gap	12	6–22
Blood urea nitrogen	16 mg/dL	7–17 mg/dL
Creatinine	1.2 mg/dL	0.7–1.2 mg/dL
Glucose	**235** mg/dL	60–110 mg/dL
Troponin I	**0.16** ng/mL	0.00–0.10 ng/mL
Creatine kinase	71 U/L	55–170 U/L

*INR* international normalized ratio

The patient was admitted to the COVID unit, and he was started on an intravenously administered, unfractionated heparin drip. Ultrasound of the lower extremity showed a non-occlusive DVT at the distal left femoral vein to the left popliteal vein. Since the patient was hemodynamically stable, tissue plasminogen activator (TPA) administration was not considered. His symptoms significantly improved with anticoagulation. The hypercoagulable panel did not show any other active risk factor for thrombotic conditions.

The patient was observed in the hospital for 3 days and then was discharged home on the enoxaparin bridge with warfarin. Since there was a high suspicion of SARS-CoV-2 infection because of exposure through the family member, the patient was instructed to follow strict COVID isolation precautions. Eight days later, his COVID-19 test also came back as positive. Post-discharge telephone calls at day 10 and week 4 revealed that the patient was appropriately responding to anticoagulation treatment and had no recurrence of his symptoms related to VTE/COVID-19.

## Discussion

The COVID-19 pandemic started in December 2019 with a collection of patients presenting with pneumonia of unknown origin connected to a seafood wholesale market in Wuhan, China [1]. The source of this presentation was identified as a novel coronavirus, later named as severe acute respiratory syndrome coronavirus-2 (SARS-CoV-2) and the disease it causes was named as coronavirus disease 2019 (COVID-19) [1]. Coronaviruses are a large group of enveloped ribonucleic acid (RNA) viruses that are distributed among animals such as pigs, bats, camels, and cats. When humans acquire these viruses, they commonly cause mild-to-moderate upper-respiratory diseases [2]. Three of these coronaviruses, severe acute respiratory syndrome (SARS) coronavirus, Middle East respiratory syndrome (MERS) coronavirus, and SARS-CoV-2 have now been known to cause severe, and often fatal disease in humans [2].

New evidence, although very limited at this point, showing venous thromboembolism (VTE) as a complication of COVID-19, has started to emerge [3]. The exact mechanism of thrombus formation can be variable, but it is postulated that COVID-19 can lead to an increase in the inflammatory response, hypoxia, immobilization, and disseminated intravascular coagulation (DIC), all of which can increase an individual’s propensity to arterial and venous thromboembolic disease [3].

Viral infections, in general, can lead to an imbalance between pro- and anticoagulant states during the course of the disease and it often involves the disruption of the vascular endothelium [4]. Various pathways involving the coagulation cascade, including elevated von Willebrand factor, cause the development of cross-linked fibrin clots. The breakdown of these clots leads to elevation of D-dimer levels and fibrin degradation product levels, both of which are associated with poor prognosis in COVID-19 patients, including the need for intensive care unit (ICU) admission, and even death [5, 6]. This activation of the systemic coagulation, along with immobility associated with bed rest, in COVID-19 patients increases the risk of VTE [3, 4, 6].

In a single-center cohort study, Middeldorp et al*.* reported objectively confirmed VTE incidence rates of 16%, 33%, and 42% at days 7, 14, and 21, respectively, among hospitalized patients with COVID-19, despite receiving thrombosis prophylaxis. This, however, was a single-center cohort study with a small sample size of 199 patients [6]. In a separate study, Klok et al*.* reported a 31% incidence of objectively confirmed VTE and arterial thrombotic complications despite standard thrombotic prophylaxis [3]. This again is a retrospective study with a small sample size of 184 patients. In another retrospective cohort study of 388 patients with COVID-19, Lodigiani et al. reported a cumulative VTE incidence rate of 21%, with half of those events occurring within 24 h of admission to the hospital [7].

Due to the lack of availability of sufficient data, the concept of whether or not to use prophylactic anticoagulation in hospitalized COVID-19 patients to improve their overall clinical outcome continues to remain controversial [4]. Klok et al. and Middeldorp et al. advise against prophylactically initiating treatment-dose anticoagulation in all hospitalized patients with COVID-19 and instead are recommending physicians to use a lower threshold for appropriate diagnostic tests for evaluation of thrombotic complications including DVT and pulmonary embolism [3, 6]. More research involving randomized, controlled clinical trials are needed to know definitely whether preemptive and prolonged treatment-dose anticoagulation leads to a favorable clinical outcome in patients with COVID-19 infection.

## Conclusion

COVID-19 continues to cause significant mortality as more data is emerging that is aiding in piecing together its perplexing pathogenicity. Scant emerging data is suggesting COVID-19’s role in increased activation of systemic coagulation and further leading to thromboembolic complications such as pulmonary embolism. Here, we presented a case highlighting this very rare presentation. For future direction, we recommend more research focusing on ethical, sound, randomized, controlled clinical trials evaluating the efficacy, safety, dosing, and duration of prophylactic anticoagulation in all hospitalized patients infected with COVID-19 in order to improve survival rates.

## Data Availability

Not applicable

## Abbreviations

VTE: Venous thromboembolism
SARS-CoV-2: Severe acute respiratory syndrome coronavirus-2
COVID-19: Coronavirus disease 2019
PCR: Polymerase chain reaction
DVT: Deep vein thrombosis
tPA: Tissue plasminogen activator
ICU: Intensive care unit
DIC: Disseminated intravascular coagulation
CT: Computed tomography
EKG: Electrocardiogram
